# Ablation of the Renal Stroma Defines Its Critical Role in Nephron Progenitor and Vasculature Patterning

**DOI:** 10.1371/journal.pone.0088400

**Published:** 2014-02-05

**Authors:** Stephanie Hum, Christopher Rymer, Caitlin Schaefer, Daniel Bushnell, Sunder Sims-Lucas

**Affiliations:** 1 Rangos Research Center, Children's Hospital of Pittsburgh, Pittsburgh, Pennsylvania, United States of America; 2 Department of Pediatrics, University of Pittsburgh School of Medicine, Pittsburgh, Pennsylvania, United States of America; National Cancer Institute, United States of America

## Abstract

The renal stroma is an embryonic cell population located in the cortex that provides a structural framework as well as a source of endothelial progenitors for the developing kidney. The exact role of the renal stroma in normal kidney development hasn't been clearly defined. However, previous studies have shown that the genetic deletion of Foxd1, a renal stroma specific gene, leads to severe kidney malformations confirming the importance of stroma in normal kidney development. This study further investigates the role of renal stroma by ablating Foxd1-derived stroma cells themselves and observing the response of the remaining cell populations. A Foxd1cre (renal stroma specific) mouse was crossed with a diphtheria toxin mouse (DTA) to specifically induce apoptosis in stromal cells. Histological examination of kidneys at embryonic day 13.5–18.5 showed a lack of stromal tissue, mispatterning of renal structures, and dysplastic and/or fused horseshoe kidneys. Immunofluorescence staining of nephron progenitors, vasculature, ureteric epithelium, differentiated nephron progenitors, and vascular supportive cells revealed that mutants had thickened nephron progenitor caps, cortical regions devoid of nephron progenitors, aberrant vessel patterning and thickening, ureteric branching defects and migration of differentiated nephron structures into the medulla. The similarities between the renal deformities caused by Foxd1 genetic knockout and Foxd1DTA mouse models reveal the importance of Foxd1 in mediating and maintaining the functional integrity of the renal stroma.

## Introduction

Development of the mature kidney involves complex interactions between the metanephric mesenchyme and the ureteric epithelium [1], [2]. Subsequently, much of the focus in the field of kidney development has centered on the interactions between these two critical cell types. However, there is an equally important cell population termed the renal stroma whose role in kidney development has not been extensively studied. The renal stroma is an embryonic cell population composed of fibroblastic spindle cells with large amounts of extra cellular matrix [3]. The stroma starts out by forming a loose domain of cells surrounding the mesenchyme that condenses around the ureteric bud [4]. As the kidney develops, the renal stroma interdigitates between the nephron progenitor caps and ureteric bud branches forming the primary renal interstitium [5]. In the mature kidney, the renal stroma gives rise to the renal capsule, interstitium, mesangium and many of the vascular supportive cells [6]. The renal stroma has also been shown to act as a rich source of vascular progenitors including smooth muscle cells, pericytes, and more recently in our own findings, a source of endothelial progenitors [7].

The renal stroma is characterized by the gene Foxd1, previously known as Brain Factor-2 (BF-2). This gene is a member of the winged-helix family of genes and serves as a transcription factor in the renal stroma. The stroma begins to express Foxd1 once the ureteric bud invades into the metanephric mesenchyme at E11, making it the earliest identifier of the renal stroma [4], [8]. Previously, it has been determined that the genetic deletion of Foxd1 disrupts the patterning and development of the kidney implicating the important role of the stroma in normal kidney development [4]. These mutant kidneys were smaller and had severe structural deformities with a high presence of fused horseshoe kidneys [9]. They also exhibited reduced branching of the ureteric bud, decreased number of nephrons, abnormalities of the renal capsule, misplaced vasculature in the renal capsule, and overall aberrant patterning of renal structures. [4], [9]. The malformation of the mutant kidneys caused by the deletion of the stroma specific gene, Foxd1, illustrates that the stroma plays a critical and involved role in normal kidney development. Furthermore, it was recently determined that the renal stroma secretes a critical factor, Fat4, that acts to aid in the differentiation of the nephron progenitors.

Subsequently, this study investigates the role of the renal stroma in patterning the developing kidney. Our results demonstrate that specific ablation of the renal stromal cell population targeted using a floxed diphtheria toxin mouse [10] in combination with a Foxd1cre mouse [11] caused many of the same phenotypic defects that were present in the previously discussed studies. However, closer examination of these developing kidneys reveals an abundance of differentiated nephron structures inappropriately formed in the medulla as well as widened and thickened nephron progenitors. Furthermore, mutants had a mispatterning of the vessels including large caliber vessels that extended into the previously occupied stromal compartments. These findings confirm that the renal stroma has a multifaceted impact on the development of multiple renal compartments. Furthermore, we saw that the stromally ablated mouse recapitulates the findings of the Foxd1 genetic knockout emphasizing that the functionality of the renal stroma probably stems from the expression of Foxd1 with little effect from the cells themselves.

## Materials and Methods

### Animals

We used the transgenic *Foxd1EGFPcre* mouse line that expresses GFP and cre recombinase in the renal stroma [11], [12]. In order to ablate the Foxd1-expressing cells, we bred *Foxd1EGFPcre* mice with Diphtheria Toxin mouse (DTA), which has a ubiquitously present Diphtheria toxin gene at the GT Rosa locus under the control of an upstream floxed-stop cassette [10]. When the DTA is bred to the *Foxd1EGFPcre*, the stop site is spliced out in the Foxd1cre expressing stromal cells causing Diphtheria toxin to be activated, selectively killing the stromal cells (producing Foxd1DTA mice). In order to permanently label the Foxd1-expressing cells, we bred *Foxd1EGFPcre* mice with GT Rosa CAG reporter mice (tdTomato) that express red fluorescent protein (RFP) in all cre positive derivatives [13]. All time-mated females were sacrificed via CO_2_ inhalation, followed via cervical dislocation. All embryos were subsequently sacrificed via decapitation. The University of Pittsburgh Institutional Animal Care and Use Committee approved all experiments.

### Genotyping

Briefly, tail clippings and/or embryonic tissues were collected and genomic DNA was isolated. Polymerase chain reaction (PCR) amplification was used to identify all genotypes. The primers used to detect the *Foxd1EGFPcre* allele were: forward 5′-TCTGGTCCAAGAATCCGAAG-3′ and reverse 5′-GGGAGGATTGGGAAGACAAT-3′ which showed a band at 450 base pairs (bp) while cre-negative mice had no band. The primers utilized to detect tdTomato were wildtype forward 5′-AAGGGAGCTGCAGTGGAGTA-3′, wildtype reverse 5′-CCGAAAATCTGTGGGAAGTC-3′, which showed a band at 297 bp, and mutant forward 5′-CTGTTCCTGTACGGCATGG-3′ and mutant reverse 5′-GGCATTAAAGCAGCGTATCC-3′ which showed a single band at 196 bp.

### Tissue collection

For paraffin sectioning the embryos were located and removed at various developmental stages at E11.5, E13.5, and E16.5. The embryos were kept whole for E11.5 and E13.5 while the kidneys were dissected out for E16.5. The samples were fixed in 4% paraformaldehyde (PFA) before being processed into paraffin wax and sectioned at 8 µm. For frozen sections, E18.5 kidneys were fixed in 4% PFA and then dehydrated in sucrose and embedded in OCT medium. Sections were cut at 10 µm on a cryostat and stored at -20°C. For whole mount immunofluorescence, organs were removed and placed into 4% PFA in PBS overnight, dehydrated through to 100% methanol, and stored at −20°C.

### Apoptois assays

Terminal deoxynucleotidyl transferase dUTP nick-end labeling (TUNEL) assays on Foxd1DTA and control (n = 3 per genotype), were performed using a Fluorescent FragEl DNA Fragmentation Detection kit (Oncogene, Cambridge, MA) on paraffin sections (8 µm) following the manufacturer's instructions. To further confirm the presence of apoptotic cells we used activated Caspase 3 antibody (Catalog #PRG7481, Fisher Scientific, Pittsburgh, PA) and co-labelled with various kidney compartment markers.

### Immunohistochemistry

For paraffin section immunofluorescence (IF), embryonic or isolated tissue sections were subjected to citrate antigen retrieval prior to being blocked in a 10% bovine serum albumin/donkey serum solution in PBS, while for frozen sections they were blocked without the antigen retrieval. Both were incubated at 4°C overnight with primary antibodies including anti-PECAM (catalog #553370, BD Biosciences, San Jose, CA), anti-NCAM (Catalog #C9672, Sigma, St. Louis, MO), anti-renin (catalog #SC27318, Santa Cruz), anti-αSMA (Catalog #A5228, Sigma), anti-Amphiphysin (catalog #13379-1-AP, Proteintech, Chicago, IL), anti-Pax2 (catalog #PRB-276P, Covance, Indianapolis, IN), anti-Foxd1 (catalog #SC47585, Santa Cruz, Dallas, TX), anti-Meis1/2 (catalog #10599, Santa Cruz), anti-Tenascin (catalog #AB19011, Millipore, Billerica, MA), anti-Jagged 1 (catalog #SC8303, Santa Cruz), anti-Lhx1 (catalog #4F2-s, Developmental Studies Hybridoma Bank, Iowa city, IA), anti-PDGFRB (catalog #04-397, Millipore) and/or anti-Six2 (catalog #11562-1-AP, Proteintech) at 1∶100 concentrations. The tissues were then washed extensively in PBS and subsequently incubated with 1∶250 concentrations of the following secondary antibodies: donkey anti-mouse Alexa Fluor-594, donkey anti-goat Alexa Fluor-488 (catalog #A11055, Invitrogen, Carlsbad, CA), goat anti-rabbit Alexa Fluor-594 (catalog #A11080, Invitrogen) or donkey anti-rat Alexa Fluor 488 (catalog #712-605-150, Jackson Immunoresearch, West Grove, PA). The sections were then extensively washed, mounted, and visualized with a Leica upright microscope (Buffalo Grove, IL). For the wholemount IF, the kidneys were rehydrated through graded methanol series to 0.1% Tween in PBS (PBST). After blocking in 10% donkey serum in PBST for 1 hour at room temperature, tissues were incubated with 1∶100 concentrations of the following antibodies: anti-calbindin (catalog #C9848, Sigma-Aldrich, St Louis, MO), anti-PECAM (catalog #553370, BD Biosciences) anti-Foxd1 (catalog #sc47585, Santa Cruz Biotechnology, Santa Cruz, CA) and/or anti-Six2 (catalog #11562-1-AP, Proteintech, Chicago, IL) primary antibodies at 4°C overnight. The tissues were then washed extensively in PBST and subsequently incubated with 1∶100 concentrations of the following secondary antibodies: donkey anti-goat Alexa Fluor-488 (catalog #A11055, Invitrogen, Carlsbad, CA), goat anti-rabbit Alexa Fluor-594 (catalog #A11080, Invitrogen) or donkey anti-rat Alexa Fluor 647 (catalog #712-605-150, Jackson Immunoresearch, West Grove, PA). The kidneys were then extensively washed, mounted, and visualized with an Olympus confocal microscope (Center Valley, PA).

### In situ hybridizations

The in situs were carried out as previously described [14]. We utilized Ret and Wnt11 to visualize the ureteric tips.

### Real time PCR

Real time PCR was performed as described previously [15]. Briefly, mRNA was extracted from snap frozen E13.5 *Foxd1DTA* and control kidneys (n = 3 per group) (Qiagen, Valencia, CA). Primers for *Foxd1* were utilized with *Gapdh* as an endogenous control (Invitrogen, Carlsbad, CA). Quantitative real-time PCR was performed on an Applied Biosystems ABI 7900 HT (Foster City, CA).

### Results and Discussion

The ablation model of Foxd1 using DTA in many ways phenocopied the knockout, suggesting that Foxd1 signaling is likely the critical factor-governing kidney patterning from the renal stroma. However, the ablation of the Foxd1 cells revealed critical extensions of the previous models including the mispatterning of the differentiated nephron structures and the developing vasculature. These findings are discussed below.

### Diphtheria toxin induced apoptosis is seen as early as E11.5 in Foxd1DTA mutant kidneys

**Figure 1:**
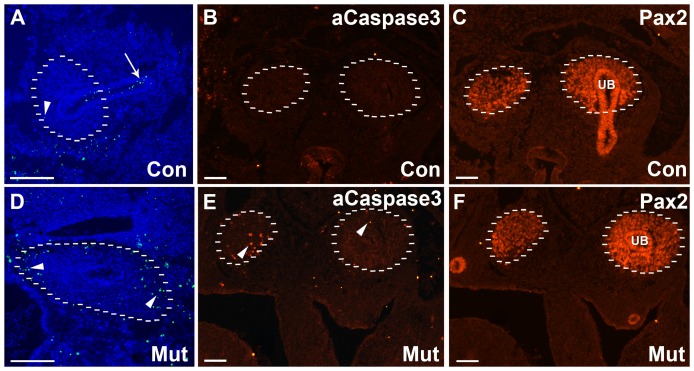
Apoptosis is up-regulated in the metanephric mesenchyme of Foxd1DTA mutants. A–C: Control E11.5 kidney showing apoptotic cells in relation to the developing mesenchyme and ureteric bud. A. Apoptotic cells are observed in the common nephric duct (arrow) however very few are seen throughout the metanephric mesenchyme (arrowhead). B–C. representative activated Caspase 3 staining showing very few apoptotic cells in the metanephric mesenchyme (dotted lines) or ureteric bud (UB) of controls as marked by Pax2 staining (C). D–F. Control E11.5 kidney showing abundant apoptotic cells via both apoptosis assay (D) and activated Caspase 3 (E) throughout the metanephric mesenchyme (arrowheads) as marked by Pax2 staining (F). Scale bar = 100 µm.

The renal stroma begins to express Foxd1 as soon as the ureteric bud invades the metanephric mesenchyme around E11. To confirm cre activity at this early time point we bred the *Foxd1creEGFP* mice with a Tdtomato reporter mouse and showed cre activity at this early time point (Figure S1). Since the Foxd1DTA mutants specifically target cells containing the gene Foxd1 for deletion, we wanted to quantify the effectiveness of the diphtheria toxin to kill these cells. In order to do this, we performed an apoptosis assay and immunohitochemistry for activated Caspase 3, we used Pax2 and Tenascin to delineate the renal linages at E11.5, E13.5, and E16.5 (Figure 1 and Figure S2). At E11.5 the apoptosis could be seen throughout the metanephric mesenchyme, an area typically occupied by the Foxd1 positive stroma. However, at later developmental stages via immunohistochemistry we determined that a significant amount of apoptosis was occurring in the Pax2 positive condensing mesenchyme, while the Foxd1 stroma was no longer apparent. This deletion of the Foxd1 positive-cells was confirmed via immunohistochemistry and qPCR, which showed a 73% decrease in Foxd1 expression at E13.5 (Figure S3) and by E18.5 the Foxd1 stroma was completely absent (data not shown). These findings confirm that the diphtheria toxin in the Foxd1DTA mutants was in fact inducing apoptosis in Foxd1 expressing stromal cells. This increase in the amount of apoptotic cells in the cortex was present as early as E11.5, showing that the effect of the diphtheria toxin is almost instantaneous to the start of the expression of Foxd1 in stromal cells (Figure 1A–B). The continuous presence of large numbers of apoptotic cells shows that this deletion of the renal stroma continues throughout the development of the kidney and not just at the initial stages (Figure 1C–F). Furthermore, this suggests that although the metanephric mesenchyme can differentiate and produce Pax2 positive renal vesicle like structures for appropriate differentiation Foxd1 stroma is required for their maintenance. This may implicate that other lineages may be able to turn on Foxd1 expression in place of the renal stroma and potentially rescue the knockout phenotype. Some references also suggest that there may be Six2/Foxd1 double expressing cells that could be a source of the Foxd1 cells that continue to undergo apoptosis in the later developmental stages [16]. However, it is unlikely that these cells are sufficient as the phenotype is still very severe in the Foxd1DTA mice. It is more likely that the structures that do occur are a result of incomplete excision rather than inappropriate expression of Foxd1.

### Deletion of the renal stroma causes severe structural kidney deformities

**Figure 2:**
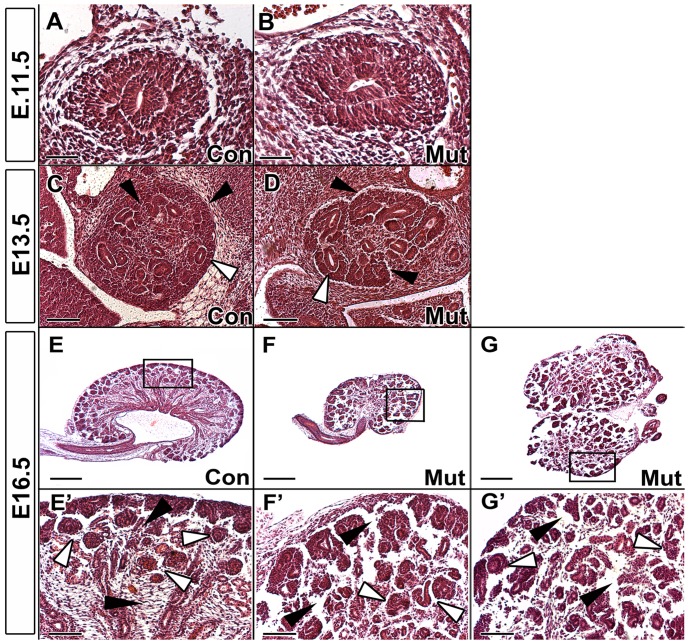
Renal stroma ablation causes morphological defects throughout development. A–B: E11.5 H&E staining shows no renal abnormalities in Foxd1DTA mutants (B) compared to controls (A). C–D: E13.5 H&E staining highlights the absence of the renal stroma in the outer cortex and interdigitating between the caps (black arrows) in mutants (D) compared to controls (C). Early signs of irregular thickening and expansion of the nephron progenitor caps are also present (white arrow). E–G: E16.5 Foxd1DTA mutant kidneys (F,G) are smaller than controls (E) and have severe structural abnormalities such as dysplastic (F) and fused horseshoe (G) kidneys. E′, F′,G′: Close up images of cortical regions of controls (E′) and mutants (F′,G′) show the absence of stroma between renal structures, especially in the spaces between the nephron progenitor caps (black arrows). Furthermore in mutants, the renal structures lack an organized structure in the cortex compared to controls (white arrows). Scale bar = A–B:50 µm; C–D:100 µm; E–G:400 µm; E′–G′:100 µm.

In order to grossly characterize the effect of the ablation of the renal stroma as the kidneys developed, we performed H&E staining at three different developmental time points (E11.5, E13.5, and E16.5). Although the deletion of stromal cells begins as early as E11.5, we saw no morphological changes in the kidney at this time point (Figure 2A–B). However, at E13.5, we clearly see the ablation of the renal stroma around the outer cortex of the kidney (Figure 2C–D), which was subsequently confirmed via real time PCR. Tangentially, we also start to see a thickening of the nephron progenitor caps (Figure 2C–D). The effects of the ablation of the renal stroma are most apparent at E16.5, at which time the mutants have severe structural defects and complete mispatterning of the renal structures. From a broad viewpoint, the mutant kidneys are much smaller compared to the controls (Figure 2E–G). Both these representative kidneys are clearly dysplastic, and one even fused together instead of forming two separate kidneys (Figure 2G). These smaller, dysplastic and fused kidneys were also characteristic of the Foxd1 genetic knockouts [9]. Focusing on the cortex of the kidney, we again see the complete ablation of the renal stroma on the outer border of the kidney as well as interdigitating between the renal structures (Figure 2E′–G′). The signs of the mispatterning of the kidney that we saw at E13.5 become even more exaggerated at E16.5. The mutants show a complete lack of organization of renal structures whereas controls have a rigid organization of renal structures lined up along the cortex (Figure 2E′–G′). Morphologically, we concluded that the ablation of the renal stroma caused large mispatterning of the renal structures and overall structural defects.

**Figure 3:**
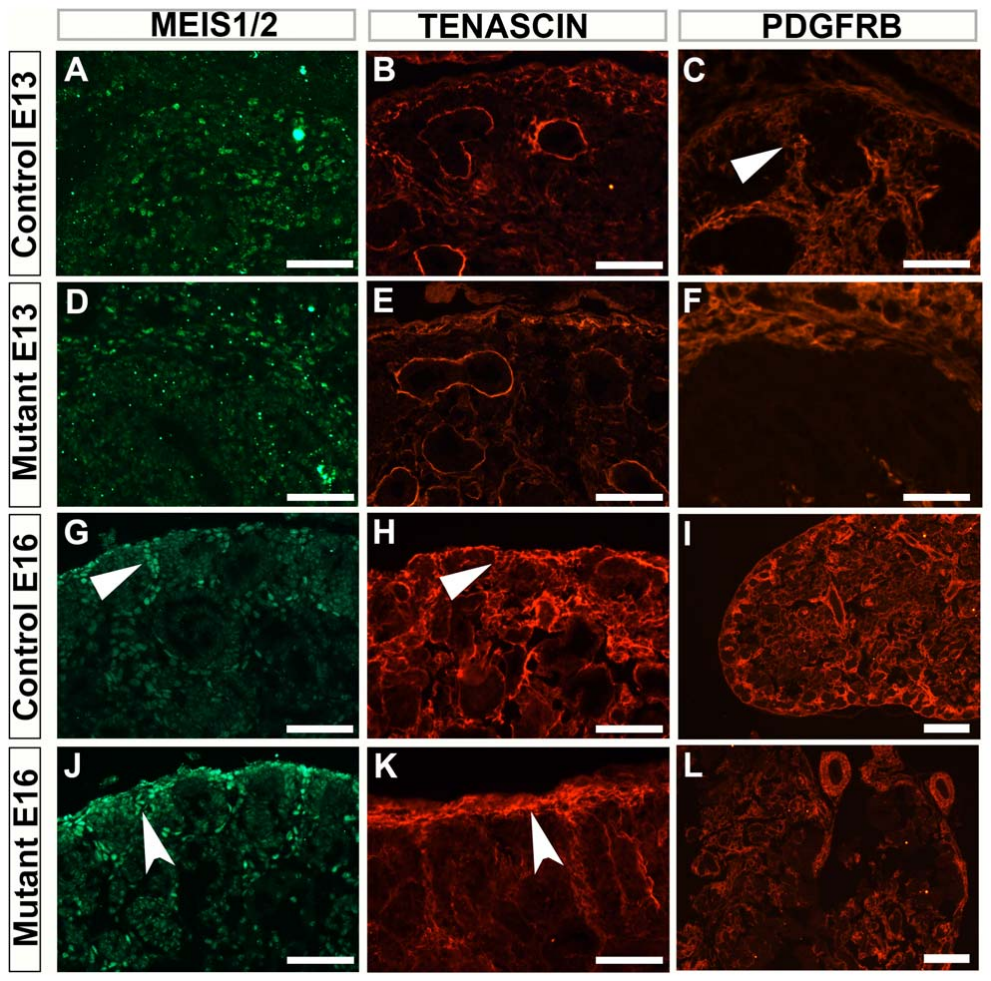
The stroma of Foxd1DTA mutants is mispatterned. A–F: E13.5 control (A–C) and Foxd1DTA (D–F) kidney sections stained with renal stromal markers. E13.5 kidneys stained with Meis1/2 (A and D), Tenascin (B and E) and PDGFRB (C and F) show that mutant samples have disorganization with a lack of stromal tissue interdigitating between the nephron progenitor units. G–L: E16.5 control (G–I) and Foxd1DTA (J–L) kidney sections stained with renal stromal markers. The lack of organization is again apparent, with a thickened capsule (concave arrow) and lack of interdigitation (arrow). Low power PDGFRB images show a fused kidney and the lack of stromal organization (I and L). Scale bars A–H and J–K = 100 µm, I and L = 200 µm.

To further evaluate the renal stroma we utilized other known markers of renal stroma, including Meis1/2, Tenascin and PDGFRB. The Foxd1 compartment of cells was clearly deleted from the Foxd1DTA kidneys however there was a clear stromal compartment with the persistence of Tenascin, Meis1/2 and PDGFRB in the periphery of the kidney. However, this stroma failed to pattern appropriately and did not send finger like projections interdigitating between the nephron forming units. Similar to what had previously been shown with the Foxd1 knockout mice the stromal compartment was not organized and thickened in places around the periphery. While in the interior of the kidney there seemed to be a loss of stromal marker staining at E13.5 (Figure 3). However, by E16.5 there was a restoration of the stromal markers (with the exception of Foxd1) however the patterning of the kidney was greatly disturbed, with the appearance of kidneys that were fused in the midline (Figure 3L).

### Expansion and inappropriate distribution of the nephron progenitors is observed in the Foxd1DTA mutants

**Figure 4:**
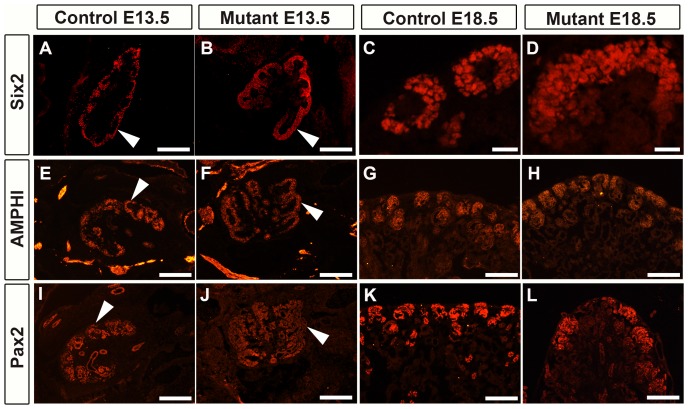
Foxd1DTA mutants have thickened and widened nephron progenitor caps. A–B: E13.5 Six2 staining reveals that mutant kidneys (B) have thicker nephron progenitors and widened progenitor caps (arrow) compared to controls (A). C–F: At E18.5, Six2 staining shows the nephron progenitor caps were thicker in the mutants (D) compared to controls (C). It is also apparent that the nephron progenitor caps experience a widening, with some mutant caps being almost twice the size (D) of the control caps (C). E–H: Amphiphysin staining reveals similar nephron progenitor thickening and disorganization of the nephron progenitors that remains apparent at E18.5 (H). I–L: Pax2 staining of the nephron progenitors confirms nephron progenitor thickening at E13.5 in mutants (J) compared to controls (I). At E18.5 the large and unorganized nephron progenitor caps are apparent in mutants (L). Scale bar = A–B:100 µm; C–F:25 µm.

We next interrogated the nephron progenitor caps via immunohistochemical staining for Six2, Amphiphysin and Pax2. Nephron progenitor caps are typically surrounded by Foxd1 positive renal stroma. We observed that the nephron progenitor caps were 2–3 cell layers thicker than the controls (Figure 4). In addition to this thickening of the caps, we noticed that the caps were wider in mutants compared to control with some mutant caps being almost twice the size of the control caps (Figure 4D). This widening and expansion of the caps started as early as E13.5 and continued to be present throughout kidney development. It was recently determined that the renal stroma secretes factors that mediate nephron progenitor proliferation and differentiation [17].

**Figure 5:**
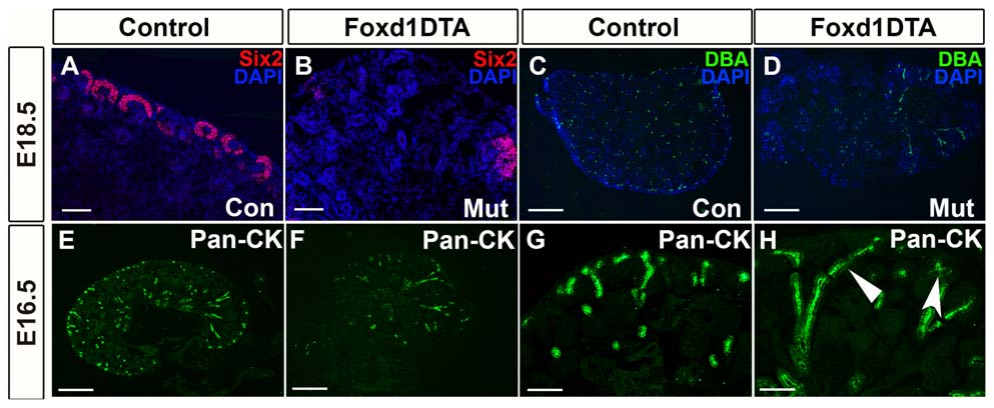
Foxd1DTA mutants have cortical regions devoid of nephron progenitor caps and ureteric branching defects. A–B: Six2 immunofluorescence staining showed that mutant kidneys (B) had gaps in Six2 expression in the renal cortex compared to the consistent line of nephron progenitor caps in controls (A). C–D: DBA staining shows an decrease in the number of ureteric branch tips in mutants (D) compared to controls. (C). E–H: Representative images of Pan-Cytokeratin (Pan-CK) staining at E16.5. Low power images show the lack of organization in the ureteric branching in mutants (F) compared to controls (E). Higher power images show that mutant ureteric epithelium (H) in some cases fails to branch (arrow), while in other disorganized branching is seen (concave arrow). Scale bar = A–B and G–H:100 µm, C–F:400 µm.

Furthermore, nephron progenitor caps normally line up around the cortex with very few, small gaps in between. Starting at E16.5, we observed large gaps in the cortex where there was no expression of Six2 nephron progenitor caps in Foxd1DTA mutants (Figure 5A–B). We next wanted to determine whether the alterations in the nephron progenitor orientation were related to changes in ureteric branching morphogenesis. It was clearly evident that there is a reduction in the amount of ureteric branching in the Foxd1DTA mice in comparison to the controls by DBA (Figure 5C–D). To further characterize the branching defect we performed Pan-cytokeratin staining at various time points and found that there was deformed branching patterns, including dilatation and branches that failed to branch (Figure 5E–H).

**Figure 6:**
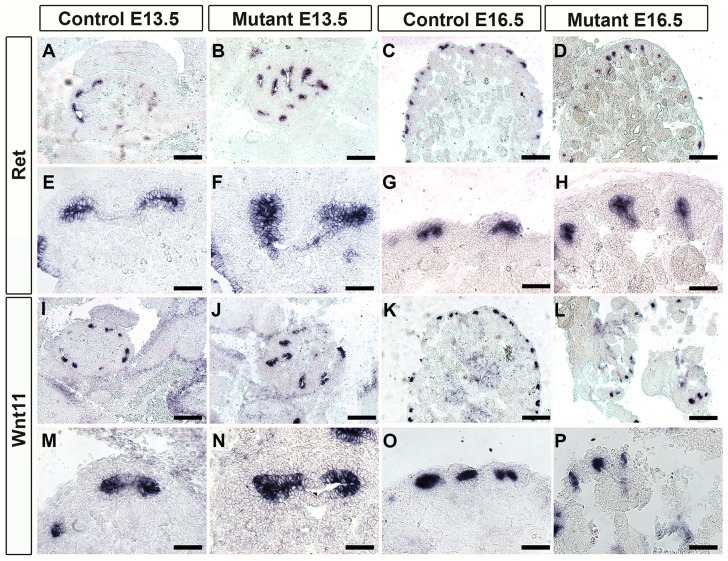
Ureteric tip markers are mis-expressed in Foxd1DTA mutants. A–H: Ret expression in Foxd1DTA mutants compared to controls. At E13.5 Ret expression is confined to the ureteric tips in controls (A and E) however in the mutants it can be seen extending down into the ureteric trunk (B and F). At E16.5 Ret expression can similarly be seen extending beyond the tips in mutants (D and H) while controls are confined to the tips (C and G). I–P: Wnt11 expression in Foxd1DTA mutants compared to controls. At E13.5 Wnt11 expression is confined to the ureteric tips in controls (I and M) however in the mutants it can be seen extending down into the ureteric trunk (J and N). At E16.5 Wnt11 expression can similarly be seen extending beyond the tips in mutants (L and P) while controls are confined to the tips (K and O). A–D and I–L Scale bar = 200 µm, E–H and M–P, Scale bar = 50 µm.

Previously, the knockout data suggested that Foxd1 mutants displayed alterations in kidney patterning including inappropriate localization of tip markers. At E13.5 we found that the tip markers Ret and Wnt11 were mislocalized down into the trunk of the ureteric epithelium (Figure 6). At later developmental time points there was more organization of the ureteric signaling although both Ret and Wnt11 still persisted beyond the tip in the mutants (Figure 6). The alterations in ureteric specification are likely a result of inappropriate signals from the renal stroma and neprogenic mesenchyme due to the inability of the nephron progenitors to differentiate[4], [17].

### Differentiated nephron structures migrate into the renal medulla

**Figure 7:**
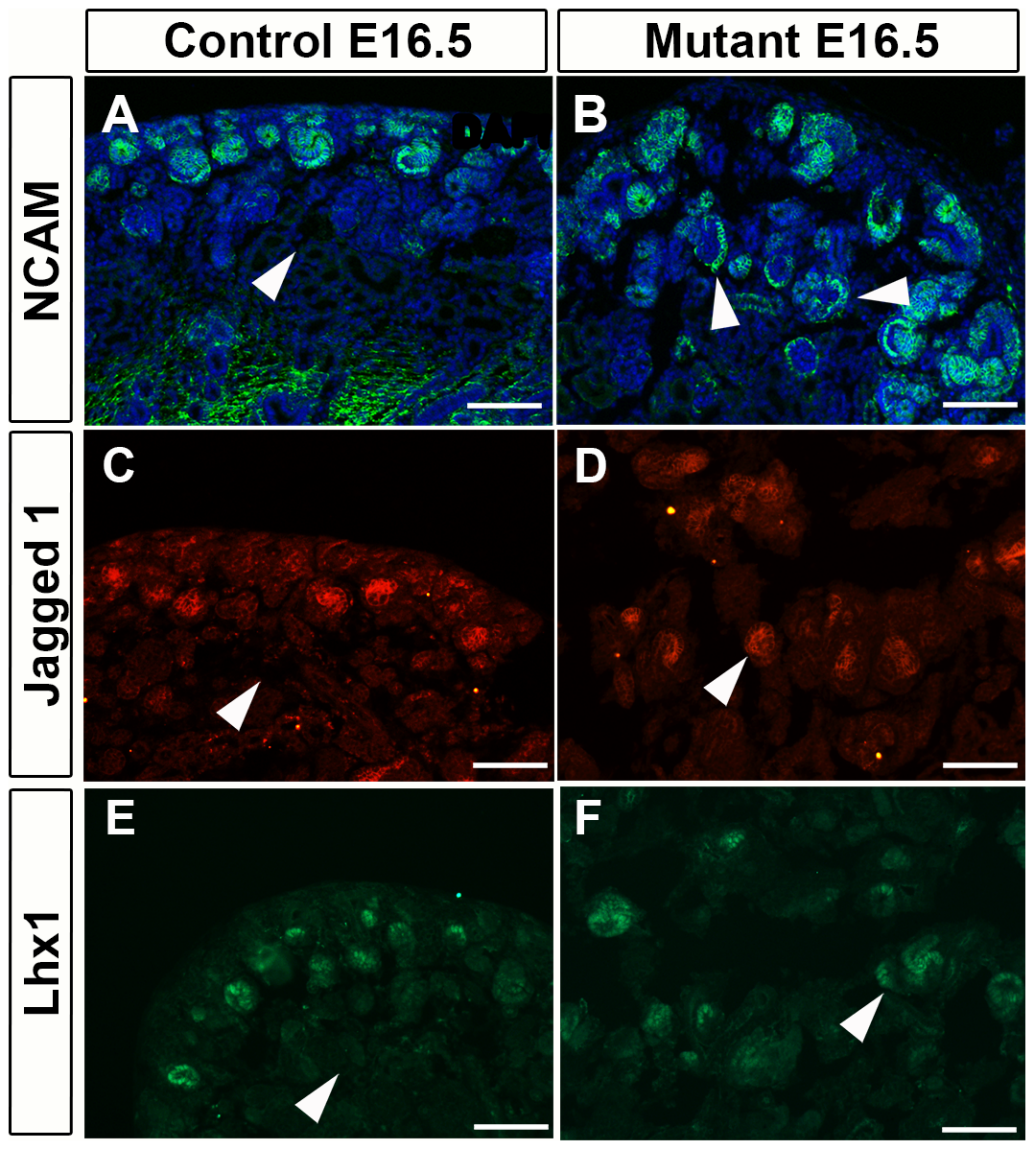
Differentiated nephron structures migrate into the medulla in Foxd1DTA mutants. A–B. E16.5 NCAM staining. In mutant kidneys (B), we observed a lack of organization in differentiated nephron structures in comparison to controls (A). Furthermore, the differentiated structures, normally in the cortex, abnormally expand into the medulla of mutant kidneys (arrows). C–D: E16.5 Jagged 1 staining. Differentiated nephron structures could again be seen deep in the medulla of the mutant kidneys (arrow). E–F: E16.5 Lhx1 staining. Differentiated nephron structures could again be seen deep in the medulla of the mutant kidneys (arrow). Scale bar = A–F:100 µm.

In order to determine the effect on differentiated nephron structures, we stained the tissue with NCAM, Jagged1 and Lhx1. Although there didn't seem to be an overall change in the amount of differentiation, the differentiated structures were highly mispatterned. Typically in controls the differentiated nephron structures form from the nephron progenitor caps in the cortex. However, in Foxd1DTA mutants, the differentiated nephron structures appeared throughout the medulla, highly unusual since nephrons always form in the cortex (Figure 7).

### Aberrant, thickened vessel formation and expansion into stromal compartments is observed in the Foxd1DTA mutants

**Figure 8:**
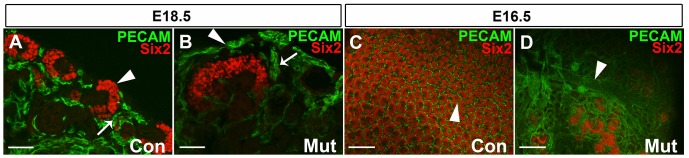
Foxd1DTA mutants have thickened vessels and abnormal patterning. A–B: E18.5 PECAM and Six2 immunofluorescence staining. Mutant kidneys (B) showed irregular vessel formation on the outside of nephron progenitor units (arrow heads) and thickened vessels between nephron progenitor caps (arrows), both of which are areas typically occupied by the renal stroma. C–D: E16.5 wholemount stains of Six2 and PECAM. Again in the mutant kidney (D), the vessels are much thicker (white arrow) than the control (C). Also, the extent of the vasculature overgrowth on the outside of the nephron progenitor cap in mutants is more apparent in the whole mount staining showing the vessels piling up thickly over top of the progenitor caps (D). A–B:50 µm; C–D:100 µm.

As the renal vasculature has been shown to be deeply embedded in the renal stroma and to give rise to a subset of vascular progenitors in the kidney we wanted to determine the effects of stromal deletion on the vasculature of the kidney [7]. In order to visualize the vasculature, we stained the tissue with the endothelial marker PECAM. We found that the majority of the vessels in mutants were thickened significantly compared to controls. Furthermore, we see the growth of the vessels into the outer most region of the cortex on the outside of the nephron progenitor caps, an area typically occupied by the renal stroma and devoid of vasculature, similar to findings in the Foxd1 genetic knockouts [9] (Figure 8). However, the Foxd1DTA mutants showed significant overgrowth of the vasculature; we observed a piling up of the vessels over the nephron progenitors and not merely a layer of vasculature overtop (Figure 8C–D). This seems to suggest unrestricted and unstructured growth of the vessels. Since the stroma is ablated, the vasculature derived from the stroma derived endothelial progenitors via vasculogenesis is also ablated [7]. The expansion of the vasculature is thus probably due to the expansion and growth of angiogenic vessels. These findings suggest that the renal stroma, in addition to being a source of endothelial progenitors, is also a regulator of angiogenic vessel growth.

**Figure 9:**
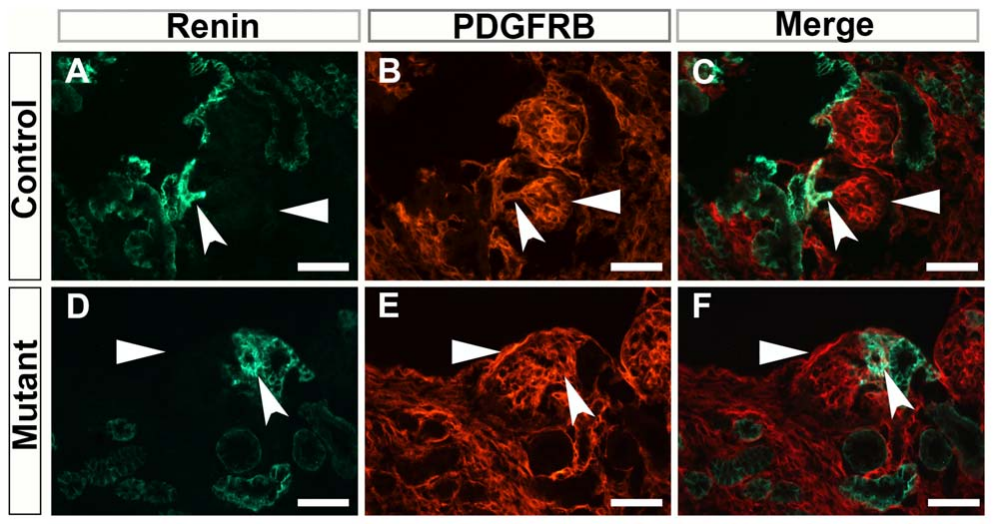
Glomeruli that form have Mesangial cells and renin-producing cells that are normally distributed in Foxd1DTA mutants. A–F: Renin and PDGFRB immunofluorescence staining within glomeruli. Renin is clearly localized in the juxaglomerular apparatus of the control (concave arrow, A and C) and mutant (concave arrow, D and F) glomeruli. While PDGFRB staining is seen localized within the mesangial cells of the glomeruli (arrows). Scale bar = 150 µm.

It has previously be shown that Foxd1 gives rise to mesangial and renin producing cells [18], [19], although there are other ex vivo transplant studies that suggest that hemogenic or external sources are able to contribute to the mesangium [18]. To evaluate the mesangial and renin producing cells and their relationship with glomeruli in this ablation model we performed immunohistochemistry for renin producing cells (that Foxd1 are known to give rise to) and the mesangial marker PDGFRB. Although the number of glomeruli that did form were extremely diminished we found that the remnant glomeruli that formed had the presence of mesangial cells as labeled by PDGFRB and that these glomeruli also had jaxtaglomerular apparatuses that expressed renin associated with them (Figure 9). The major difference was the localization of the glomeruli that formed. Glomeruli were localized throughout the entire kidney for Foxd1DTA mutants even on the very periphery of the cortex, suggesting a lack of organization and elucidating the loss of the Foxd1 stroma to pattern the metanephric mesenchyme.

From these findings we hypothesize that the alterations in kidney compartments (including vascular and nephron progenitors) seen in the Foxd1DTA mutants are likely to be as a result of spatial restriction caused by the physical presence of the Foxd1 stroma or from molecular signals that are being produced by the renal stroma to pattern the developing compartments. It is likely that a combination of these two processes is critical for normal compartmentalization of the developing kidney. However, due to the similarities with the Foxd1 knockout mice phenotype it is likely that it is the molecular signals sent from the renal stroma that are paramount to the normal formation of the kidney.

## Supporting Information

Figure S1 **Foxd1creEGFP is active at E11.5 in the kidney.** A–B: Wholemount image of E11.5 (A) and E13.5 (B) Foxd1creEGFP kidney bred with a tdTomato reporter mouse. A. At E11.5 the stroma is still primitive and can be seen throughout the metanephric mesenchyme (yellow dotted line). B. At E13.5 the Foxd1 positive cells can be observed as a honeycomb pattern which would interdigitate between the forming nephron progenitor units.
(TIF)

Figure S2 **Apoptotic cells are still present at E13.5 and E16.5 in nephron progenitors.** A–H: E13.5 assessment of apoptosis in nephron progenitors and stroma of Foxd1DTA mutants. In the control (A–D) few apoptotic cells are seen at E13.5. However, in the Foxd1DTA mutants apoptotic cells are clearly evident in the nephron progenitors (arrows) while they are largely absent from the Tenascin positive stroma (concave arrows). I–P: E16.5 assessment of apoptosis in nephron progenitors and stroma of Foxd1DTA mutants. By this stage activated Caspase 3 cells are present in the Foxd1DTA mutants in both the Tenascin and Pax2 positive cells. A, E, I–P Scale bar = 100 µm, B–D and F–F scale bar = 50 µm.
(TIF)

Figure S3 **Down-regulation of Foxd1 expression reconfirms renal stroma ablation.** A–B: Wholemount kidney stains merged for Six2 and Foxd1. A′–B′: Isolated Foxd1 wholemount staining. There is a large decrease of Foxd1 expression in mutants (A-A′ arrows) compared to controls (B-B′ arrows). Tangentially, the nephron progenitor caps also show compete disorganization, malformation, and thickening in mutants (A) compared to controls (B). C–D: qPCR of Foxd1 showed a 73% down-regulation of Foxd1 expression in mutants compared to controls. (C–D). This together with the decrease in Foxd1 immunofluorescence staining reconfirms the deletion of Foxd1-positive renal stroma in the Foxd1DTA mutants.
(TIF)
