# Supplementary figures and images for: Ablation of the Renal Stroma Defines Its Critical Role in Nephron Progenitor and Vasculature Patterning
Source: PLoS One. 2014 Feb 5;9(2):e88400. doi: 10.1371/journal.pone.0088400 (PMC3914987; doi:10.1371/journal.pone.0088400)

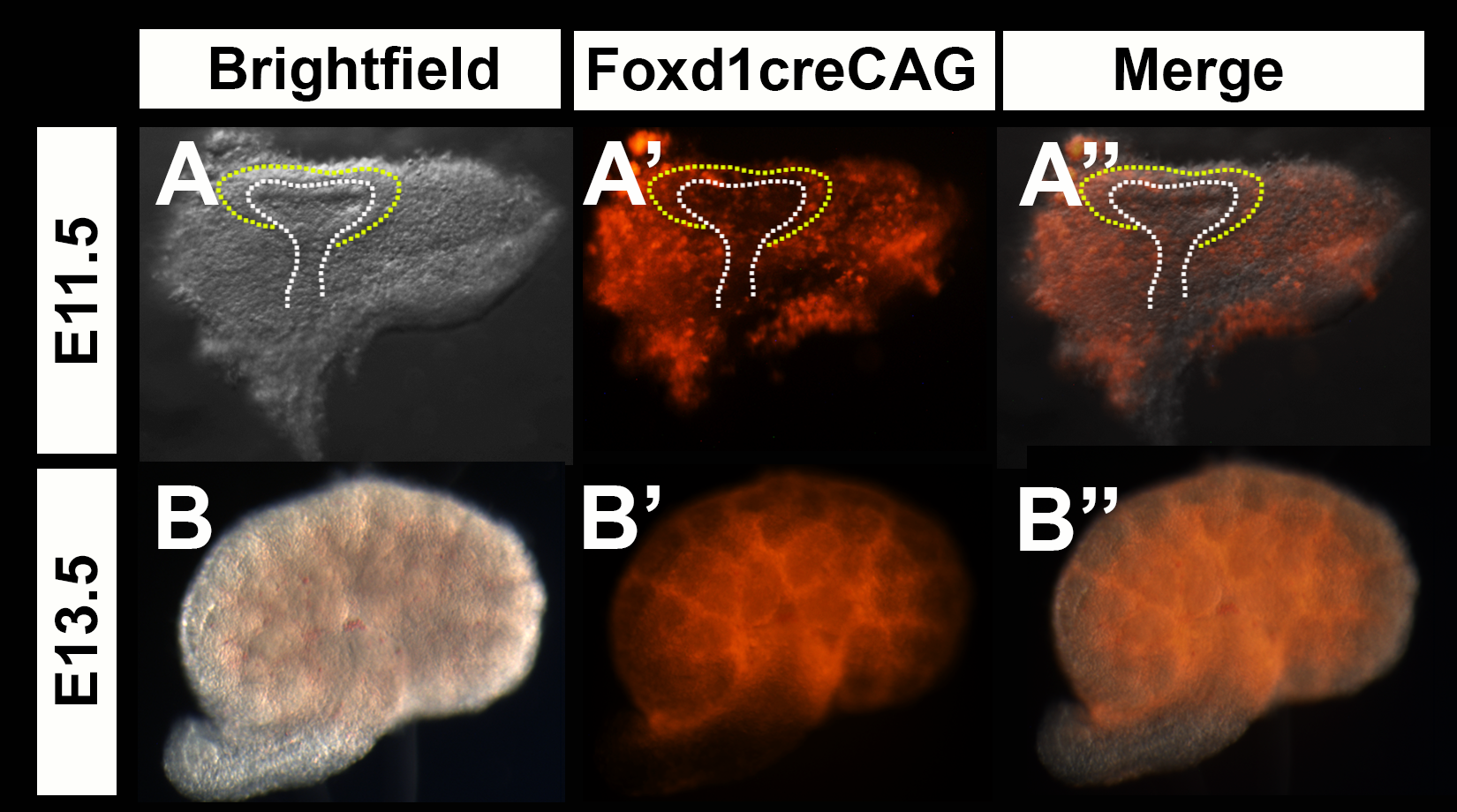

Supplement: Figure S1 — Foxd1creEGFP is active at E11.5 in the kidney. A–B: Wholemount image of E11.5 (A) and E13.5 (B) Foxd1creEGFP kidney bred with a tdTomato reporter mouse. A. At E11.5 the stroma is still primitive and can be seen throughout the metanephric mesenchyme (yellow dotted line). B. At E13.5 the Foxd1 positive cells can be observed as a honeycomb pattern which would interdigitate between the forming nephron progenitor units. (TIF) [file pone.0088400.s001.tif]

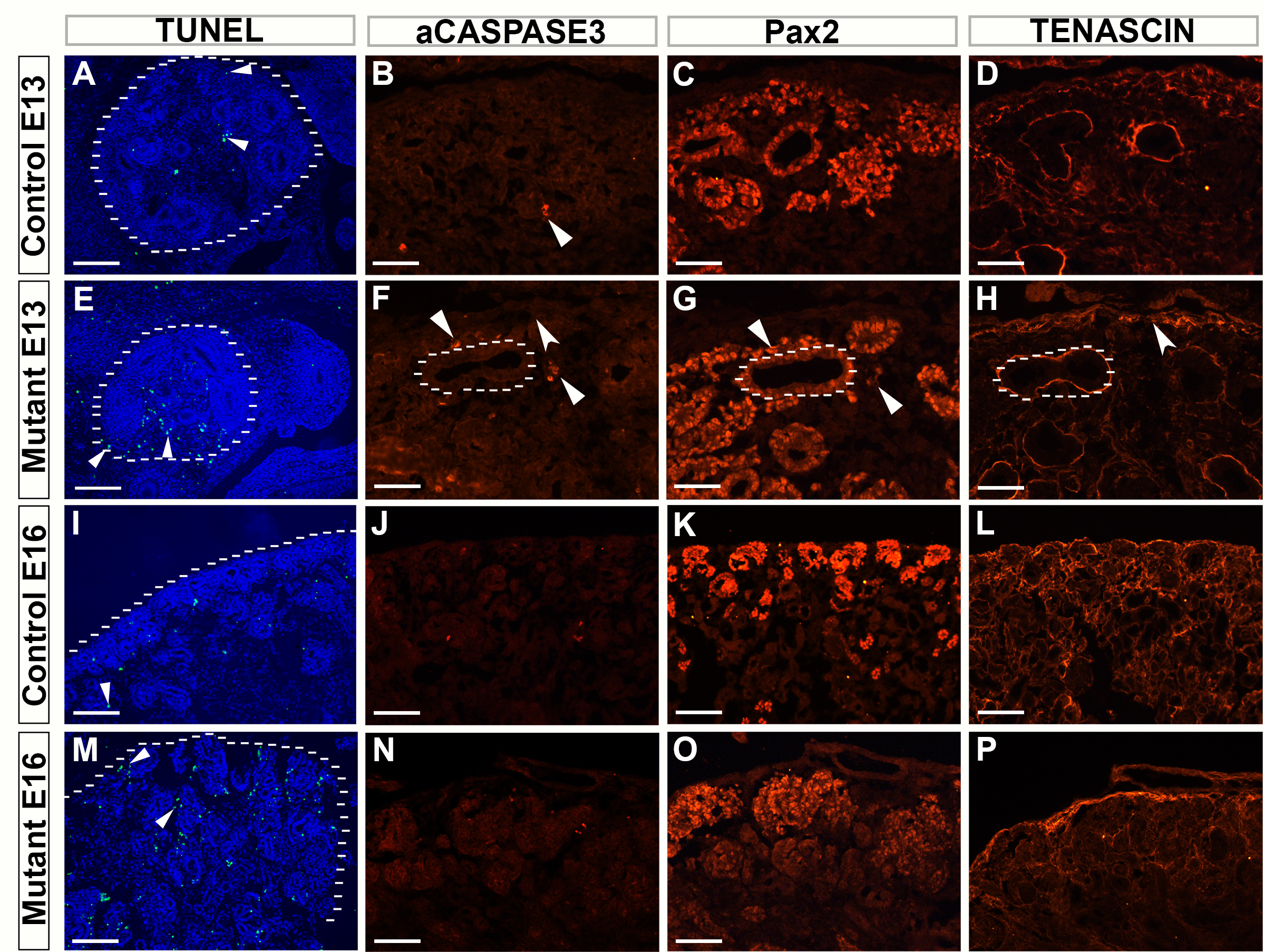

Supplement: Figure S2 — Apoptotic cells are still present at E13.5 and E16.5 in nephron progenitors. A–H: E13.5 assessment of apoptosis in nephron progenitors and stroma of Foxd1DTA mutants. In the control (A–D) few apoptotic cells are seen at E13.5. However, in the Foxd1DTA mutants apoptotic cells are clearly evident in the nephron progenitors (arrows) while they are largely absent from the Tenascin positive stroma (concave arrows). I–P: E16.5 assessment of apoptosis in nephron progenitors and stroma of Foxd1DTA mutants. By this stage activated Caspase 3 cells are present in the Foxd1DTA mutants in both the Tenascin and Pax2 positive cells. A, E, I–P Scale bar = 100 µm, B–D and F–F scale bar = 50 µm. (TIF) [file pone.0088400.s002.tif]

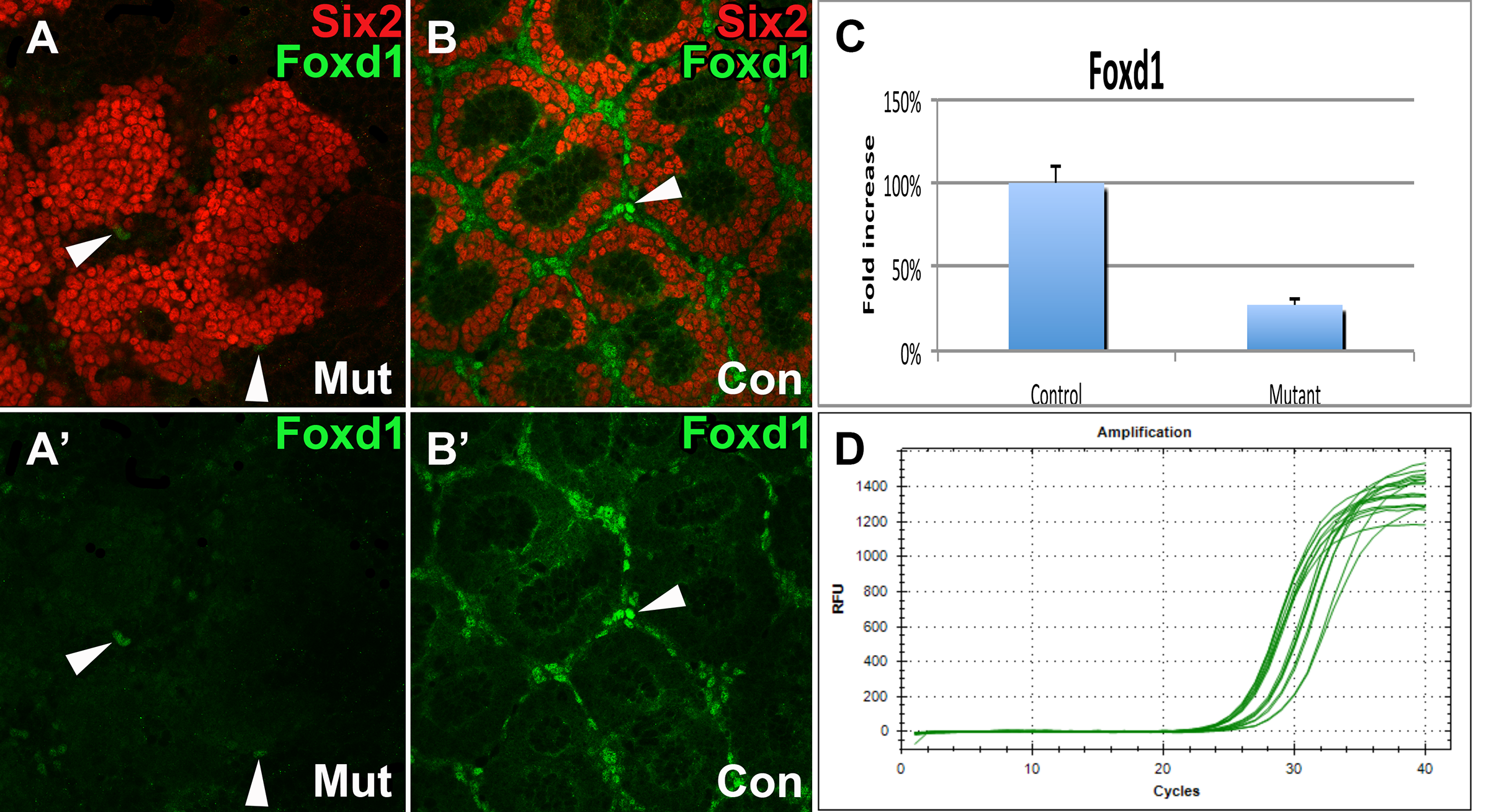

Supplement: Figure S3 — Down-regulation of Foxd1 expression reconfirms renal stroma ablation. A–B: Wholemount kidney stains merged for Six2 and Foxd1. A′–B′: Isolated Foxd1 wholemount staining. There is a large decrease of Foxd1 expression in mutants (A-A′ arrows) compared to controls (B-B′ arrows). Tangentially, the nephron progenitor caps also show compete disorganization, malformation, and thickening in mutants (A) compared to controls (B). C–D: qPCR of Foxd1 showed a 73% down-regulation of Foxd1 expression in mutants compared to controls. (C–D). This together with the decrease in Foxd1 immunofluorescence staining reconfirms the deletion of Foxd1-positive renal stroma in the Foxd1DTA mutants. (TIF) [file pone.0088400.s003.tif]
